# Benefits of group cognitive remediation therapy in anorexia nervosa: case series

**DOI:** 10.1007/s40211-016-0177-y

**Published:** 2016-03-04

**Authors:** Kate Tchanturia, Emma Larsson, Amy Brown

**Affiliations:** 1 Department of Psychological Medicine, Institute of Psychiatry, Psychology and Neuroscience, King’s College London, De Crespigny Park, SE5 8AF PO59, London, UK; 2 Eating Disorders National service, South London and Maudsley NHS Foundation Trust, London, UK; 3 Illia State University, Tbilisi, Georgia

**Keywords:** Anorexia nervosa, Cognitive remediation therapy, Cognitive flexibility, Motivation, Cognitive training, Group therapy, Anorexia nervosa, Kognitive Remediationstherapie, Kognitive Flexibilität, Motivation, Kognitives Training Gruppentherapie

## Abstract

**Background:**

Cognitive remediation therapy (CRT) is a treatment targeting cognitive difficulties in psychiatric disorders. CRT has been used with patients with severe anorexia nervosa (AN) in individual and group formats. Research of group CRT in AN is limited.

**Methods:**

Evaluation of a series of CRT groups delivered in inpatient and intensive daycare services was performed. Participants’ self-reported cognitive flexibility and central coherence, as well as motivation to change were assessed pre- and post-group. Additionally, patients’ evaluative feedback was collected after completion of the group.

**Results:**

There was a significant improvement in the patients’ self-reported cognitive flexibility and bigger picture thinking, as well as in their self-efficacy to change. The feedback questionnaires highlighted that patients found the sessions useful and reported confidence in using some of the skills and strategies they learnt in the group.

**Conclusion:**

In line with evidence from small-scale reports, this larger scale case series study indicates that group CRT leads to increased flexibility and bigger picture thinking, as well as improved confidence in the ability to change for participants. CRT in a group format seems to be a practical and helpful intervention for patients with AN in intensive treatment programmes.

## Abstract (de)

**Grundlagen:**

Die Kognitive Remediation (Englisch: Cognitive Remediation Therapy, CRT) zielt auf die Behandlung kognitiver Schwierigkeiten bei psychiatrischen Störungen. CRT wurde bisher bei Patienten mit schwerer Anorexia Nervosa (AN) im Individual- und Gruppenformat eingesetzt. Die Forschung zur CRT im Gruppenformat bei AN ist jedoch begrenzt.

**Methodik:**

Es wurden mehrere CRT-Gruppen im stationären und tagesklinischen Setting evaluiert. Selbstberichtete kognitive Flexibilität und zentrale Kohärenz sowie Änderungsmotivation wurden vor und nach der Gruppenintervention erhoben. Außerdem wurden die Teilnehmer nach Beendigung der Gruppe gebeten, ein Feedback zu verfassen.

**Ergebnisse:**

Es zeigten sich signifikante Verbesserungen in kognitiver Flexibilität, zentraler Kohärenz und Änderungsmotivation. Die Patienten gaben zudem an, die Sitzungen als nützlich empfunden zu haben und zuversichtlich zu sein, einige der erlernten Fertigkeiten und Strategien auch im Alltag umzusetzen.

**Schlussfolgerung:**

In Übereinstimmung mit kleineren Fallstudien legt die aktuelle, größer angelegte Fallserie nahe, dass die Teilnahme an einer CRT-Gruppe mit gesteigerter kognitiver Flexibilität und verbesserter zentraler Kohärenz sowie mit einer Zunahme der Änderungsmotivation einhergeht. CRT im Gruppenformat erscheint somit als machbare und hilfreiche Intervention im Rahmen intensiver Behandlungsprogramme für schwere AN.

## Introduction

Anorexia nervosa (AN) is a life-threatening psychiatric disorder with high levels of functional and social impairment [1]. The high morbidity and mortality rates associated with the illness [2, 3] emphasize the critical need to develop tailored, evidence-based treatment interventions. Over the last few years, focus has started to shift from behavioural eating disorder symptoms to the “bigger picture” of recovery; addressing broader goals for patients and providing strategies for reconnecting with life outside of their eating disorder.

In the context of wellbeing and recovery, cognitive factors play a critical role. During the last 3 decades, research into cognitive styles [4, 5] and its importance in treatment have been actively explored. Cognitive training has been found to hold promises for personalized care of individuals in numerous mental illnesses, as well as been a target in the development of behavioural interventions [6]. A key clinical intervention targeting thinking styles, with growing empirical support, is cognitive remediation therapy (CRT; for review see [7]). CRT is an easy to deliver, manualised treatment, tailored to target problematic thinking styles (for details of the manual see http://www.katetchanturia.com-publications; for current research evidence see [8]). CRT addresses the processes of thought rather than content. The treatment package includes a large number of specific cognitive exercises developed to practice cognitive flexibility, attention switching, multitasking and bigger picture thinking, as well as including tasks where a less perfectionistic (estimation) approach is required. After completing cognitive tasks jointly with the therapist, reflection takes place—this is a very important element as it helps patients to see the links between the exercises and examples in their daily life [8]. CRT is delivered in a motivational way and the collaborative approach creates easy dialogue between patients and therapists.

The individual format of CRT is actively researched in the field of eating disorders and even more in other disorders (e.g. see systematic reviews in patients with traumatic brain injury [9], epilepsy [10], attention deficit [11] and schizophrenia [12]). Evidence to date shows that CRT, delivered in an individual format, is beneficial to engage less-motivated patients in treatment [13]. Crucially, it improves cognitive performance and some evidence suggests that it contributes to improvements in quality of life. Moreover, patients and therapists rate the intervention highly beneficial (for systematic review of the literature in eating disorders see [7, 8]). In psychosis literature, it has been clearly demonstrated in randomized treatment trials that patients who went through remediation programmes had significantly better employment rates and better functioning compared to those who did not receive CRT [14]. Whereas in the field of eating disorders more research is needed to explore associations between improved cognitions and quality of life [15, 16].

Unlike individual CRT for AN where several case series and three published randomized treatment trials (RCT) are available [16–18], CRT in a group format is less systematically researched, although small pilot studies have been conducted with positive results [19–24]. Since the first pilot work of CRT in a group format [24], five peer-reviewed studies have been published to our knowledge, with significant differences between the studies (in terms of number of sessions, number of participants, outcome measures). Importantly, they have all presented potential benefits of the intervention.

The original study conducted by Genders and Tchanturia [24], investigated the effect of group CRT on patients’ self-reported motivation and cognitive styles after four weekly sessions. Significant changes were observed for the participants’ motivation, in terms of their self-rated ability to change. Similarly, Pretorious and collegues investigated the effect of four weekly session of group CRT in an adolescent sample. The authors did not find significant changes, however their effect sizes indicated small improvements on the Cognitive Flexibility Scale (CFS) and the participants’ importance to change (Motivational Ruler subscale) [22].

Two studies investigated the effect of group CRT over ten sessions [21, 23]. It was observed that participants appeared more aware of individual cognitive deficits after completing the group and noticeable improvements were found on performances on some tasks during the course of the treatment [23]. Additionally, the authors reported positive evaluations from patients and facilitators, and patients were able to reflect on their thinking style more post-treatment, as well as tolerating own mistakes [21]. Due to the small sample size no statistical analysis was conducted.

Group CRT for AN in a guided self-help format delivered in collaboration with carers was recently explored in a pilot study [19]. The study reported overall positive feedback from qualitative interviews, as well as an improvement on both patients and mothers neuropsychological performance after treatment with small to large effect sizes. In a French study—[20], Asch and colleagues reported improved scores on various neuropsychological assessments after 10 weekly group sessions of CRT for the two remaining inpatients participating in the study. Across the literature evaluating CRT in a group format, there is agreement on the positive feedback received from patients, clinicians and carers [25]; however, more formal evaluation and research is needed.

The key purposes of this study were (a) to examine a larger case series of CRT groups delivered in adult inpatient and daycare services for AN with self report measures, (b) to evaluate feedback from study participants and (c) discuss possible future developments and how to generate further evidence for group CRT work.

## Methodology

### Participants

Participants had a The Diagnostic and Statistical Manual of Mental Disorders, Fifth Edition (DSM-5) diagnosis of AN and were part of the intensive care programmes in the South London and Maudsley NHS Foundation Trust (SLaM). All participants in this study were females. Ethical approval for the study was granted by the local ethics committee (reference 05/Q0706/315). There were no specific inclusion/exclusion criteria as the sample was from a clinical programme including all patients who were females, receiving treatment in the specialist eating disorder service with a diagnosis of AN on admission. Participants at all stages of the treatment programme and recovery were invited to attend the group. All participants received other clinical input during the treatment, including nutritional, medical, individual therapy and therapeutic groups as part of the inpatient programme.

### Self-report measures

#### Detail and Flexibility Questionnaire (DFlex) [26]

A 24-item questionnaire measures cognitive rigidity and attention to detail on a 6-point Likert scale ranging from one (strongly disagree) to six (strongly agree). The measure is scored across two subscales, cognitive rigidity (e.g. “When others suggest a new way of doing things, I get upset or unsettled”) and attention to detail (e.g. “I can get hung up on details when reading rather than understanding the gist”), where the clinical cut off for the cognitive rigidity subscale is 53 and above, and greater than 44 on the attention to detail subscale. The authors reported high internal reliability with a Cronbach’s alpha of 0.88 and 0.91 for the “attention to detail” and “cognitive rigidity” subscales, respectively. The measure also displayed a good construct validity (moderate to large correlations) and discriminant validity when compared with similar subscales of the Autism Quotient [27]. In the current study, the overall Cronbach’s alpha coefficient was 0.94.

#### Cognitive Flexibility Scale (CFS) [28]

A 12-item questionnaire assesses the participants’ perception of the options and alternatives available to them in everyday situations. The scale gives a total score between 0 and 72, with higher scores representing greater cognitive flexibility. The authors reported good test–retest reliability by displaying a correlation quotient of 0.83 in a student sample [28]. The measure has been less used in clinical populations. In the current study, the overall Cronbach alpha coefficient was 0.54.

#### Motivational Ruler (MR) [29]

A 2-item self-report measure assesses participants’ importance and ability to change. The scale ranges from 0 to 10 with higher scores indicating greater importance or ability, respectively. This scale was used in previous evaluations of the individual and group work.

#### Feedback questionnaire

The patients were also given a feedback form in the last session. The questionnaire first asked patients to rate on a 5-point Likert scale how much they enjoyed the sessions, how useful the sessions were, whether they had learnt any new skills and what they thought about the length of the group. In addition, there were three open-ended questions asking patients what they liked most about the sessions, what could be improved and other groups they had attended.

## The cognitive remediation therapy groups

The outcome data was collected from 20 CRT groups facilitated in inpatient and intensive daycare services for patients with AN in 2010–2015. The typical admission criteria for these programmes are low weight and psychological problems which are difficult to manage with only weekly outpatient appointments/sessions. CRT groups are also named “Flexibility workshops”—a title suggested by patients in previous groups with the rationale that workshops sounded more engaging. CRT groups and “Flexibility workshops” have the same content but for the consistency with existing literature we will use the term CRT groups in this manuscript.

All sessions include the following elements: psychoeducation, practical exercises, reflection and discussion, as well as planning of homework tasks and challenges to try outside the group. The facilitators aim to take a motivational and collaborative stance, for example, facilitators take part in all the group exercises and discussions. We explore the different thinking styles of the group members, highlighting that there are no right or wrong ways of thinking, but pros and cons for each. The first session involves information and current research findings about the brain, thinking styles and what research tells us about cognitive styles in eating disorders. The following sessions focus on bigger picture thinking, switching and multitasking. The last session involves creating mind maps (summarizing the group) and relating the group content to the bigger picture of recovery. The groups are designed to be delivered by multidisciplinary staff members with two facilitators per group (at least one from the Psychology team).

The groups have 4–6 sessions; currently the four sessions group format is offered in our intensive daycare services and six sessions in inpatient services. The patients in earlier runs of the group [24] received only four sessions at the inpatient ward, which later was extended to six group sessions, in response to feedback from patients and facilitators. The current study includes all group lengths to be able to evaluate the group in general (group sessions are described in detail in the book [8] and clinicians manual [http://www.katetchanturia.com]).

### Procedure

The group ran once a week over 4–6 weeks, and each session was 45–50 min in duration. Patients completed the questionnaires before the beginning of the first session and again at the end of the final session. The feedback questionnaire was completed at the end of the group.

### Data analysis

Paired t-tests were conducted on the scores from each of the outcome measures from the first and final sessions using SPSS version 22. Cohen’s d effect sizes were computed for the pre- and post-measures. There was a 39.5 % drop out from the group; this was due to various reasons (e.g. discharged from clinic prior to the end of the group, failed to complete the last set of questionnaires). Out of the total participants, 21 participants had not completed the questionnaire at the beginning of the group. Thus, only the remaining 98 participants who completed both time 1 and time 2 of at least one outcome measure (at the first and last sessions) were included in the analysis. Number of participants varies across outcome measures (DFlex = 42, CFS = 77, MR = 98) due to variation of outcome measures used in the clinical audit over time.

### Qualitative data

Thematic analysis as described by Braun and Clarke (2006) was used to analyse the feedback questionnaires and to identify themes. First and second authors coded and organized responses and created tables with most common themes. Calculations were made on frequencies to demonstrate the proportion of patients highlighting the same point in the open-ended questions.

## Results

### Patient characteristics

The mean age of the participants was 26.3 (range 17–59). The mean body mass index (BMI) at the first session was 15.6 (range 11.6–19.4). Of the individuals 7 % were in the severe and enduring range of AN (BMI < 13); 32 % had severe AN (BMI between 13 and 15); 40 % were in the AN range (BMI of 15–17.5) and the remaining 21 % of the individuals were in the underweight range (BMI between 17.5 and 20). Participants at all stages of the treatment programme were invited to take part in the groups, thus a proportion of patients would have restored weighed before starting CRT. Overall weight gain was significant (*p* < 0.001, d = 0.3) presenting a mean BMI of 16.1 at the last session.

The majority of the patients were diagnosed with either restrictive AN (70 %) or AN binge-purge (22 %), with the remaining participants having a diagnosis of, EDNOS or atypical AN. The mean age of onset was 16.8 years (SD = 4.9), and the mean duration of illness was 9.7 years (SD = 7.5). Of the patients 42 % received individual CRT in addition to the group.

### Cognitive flexibility and motivation to change

Mean scores, standard deviations and results from t-tests, including effect sizes, on all outcome measures after the first and last sessions are displayed below in the Table 1.

**Table 1:** Mean scores, standard deviations of the measures before and after group CRT

		First session		Final session		*p*	*d*
Measures	*N*	Mean	SD	Mean	SD		
DFlex Cog Rig	42	55.14	9.76	51.76	9.49	0.005*	0.36
DFlex Attn Det	42	52.69	10.19	49.07	9.57	0.008*	0.37
CFS	77	44.61	9.33	46.23	8.37	0.08	0.18
MR importance	98	7.97	2.25	8.05	2.14	0.65	0.04
MR ability	98	4.97	2.62	5.86	2.69	0.001**	0.34

N number of the participants, Dflex detail and flexibility questionnaire, cognitive rigidity and attention to details subscales, CFS Cognitive Flexibility Scale, MR motivational ruler, importance to change and ability to change subscales

* significant at 0.01

** significant at 0.001

There were significant differences in the pre- and post-group scores on both subscales of the DFlex (rigidity and attention to detail) and MR ability to change.

### Patient feedback

**Fig. 1:**
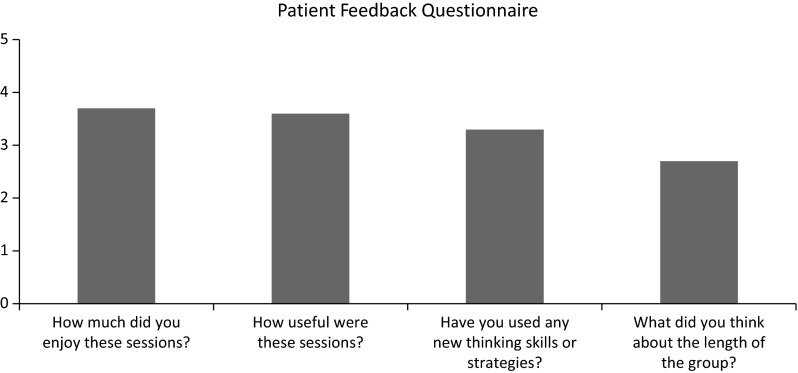
Mean scores on the feedback questionnaire given after the final session. Participants were asked to provide score on a Likert scale on each question

Figure 1 displays patients’ mean scores on the feedback questionnaire. The means suggest that the patients found sessions useful, enjoyable and have used some skills and strategies learnt in the group. When asking about the length of the group, the mean suggests that the patients thought it was “just right”, although approaching the group being “too short”.

Patients’ feedback was collected in three open-ended questions in both inpatient and daycare programmes in order to further improve group content and delivery. Feedback is summarized in Tables 2 and 3. Due to large amounts of qualitative data, we have provided main themes and some illustrative examples from the patients’ responses on the open-ended questions.

**Table 2:** Synthesis of feedback from patients: CRT elements patients liked the most are organized in themes with examples

Interactive and practical elements	Knowledge, reflection and relating it to real life	Approach to group/relaxed atmosphere	Sharing	Group facilitators
Out of the 54 responses in this theme, 37 % of the patients commented on games and interactive elements of CRT as a positive aspect of therapy:	From the 18 comments in this category, responses included learning about different thinking styles (44 %) and real-life applications (39 %)	All eight comments in this category highlighted the relaxed atmosphere (100 %). The majority of the patients felt that this group was very different from other groups which are more focused around illness symptoms. Examples:	There were 16 comments which included sharing thoughts and experiences with the group. Examples:	About 35 % of responses in both programmes included comments about group facilitators (facilitators names removed). Examples:
	Tasks relate to real life. They are objective but not subjective		The “safe space” given for people to talk about rules/routines. Also hearing suggestions from other people including group facilitators	They were interesting and also fun. I learnt things about myself. The staff were lovely
Games and tasks- kept me from switching off	Learning about my particular thinking pattern and recognizing ways to change them			
				Session leaders were very knowledgeable and welcomed our thoughts and feelings. They were very understanding and gave us a very clear idea about what expectations we could reasonably have as a result of the workshop
		Relaxed approach, combination of having plans but seeming open to discussion	Sharing ideas with peers. Provision of “food for thought” and reflection	
	Applying things learnt to our eating habits			
		The approach to the group and the atmosphere it created. People felt comfortable to input their thoughts		
				It was brilliant! Very engaging every week
		The mixture of light-hearted exercises which		
		could lead to some thought-provoking, deep discussions. Felt a “safe” place to talk, non-judgmental. Open yet challenging fixed behaviour/thinking styles		
The light heartedness sometimes with exercises and games				
	Thinking about introducing flexibly into my daily life. Not forced to talk. Liked the “quotes” handout, with positive affirmations on it			
The games that got me trying new things				
Games—positive distraction and example of flexibility				
	The gaining of a larger perspective on things and being able to zoom out and see the bigger picture			
Of the patients’ feedback 30 % included positiveaspect of interactive elements of CRT:				
The incorporation of tasks, it was not all passive. The group was small, therefore it was easy to talk and contribute				
Doing activities took my mind off of thoughts after lunch				
Being practical and being able to see links between behaviour and thought patterns. I liked coming away with something practical to work on during the week				
The practical activities we did in the session, the different tasks we were given for homework, interacting in a group. Sharing 30 % of the responses had component of having fun:				
Interactive games and visual images, well organized, fun, not too serious				

**Table 3:** Synthesis of patients’ feedback on suggested improvements, presented in themes with examples

Duration	Content/activities	Homework/handouts	Application
From 28 responses, 89 % of patients commented that they felt it would have been beneficial to have more. Examples illustrating the theme: More sessions and info regarding CRT Run them more often and longer More sessions please—8 instead of 4 It needs to be carried out on a more regular basis e.g. 8 week-cycles. Could also set people specific challenges each week Extend length of program, handbook of useful tips and practical tasks	This category varied but from 16 comments, the common themes were a larger variety of practical elements (38 %) and more practical elements in the group (43 %): A different variety of tasks if it is with the same people so it doesn’t feel repetitive More interactive things Do more practical tasks. Maybe bring in something from home life, college or work and try to solve the problem in the group using tasks discussed in sessions i.e. multitasking making an important phone call I enjoyed a wide range of activities involving mixed media, such as videos, and doing group activities. Perhaps just carry on with doing these	From 14 comments, about 40 % of the patients felt the need for more homework, examples from this category from patients quotes include: More homework, reminder of choice, include real-life situations More information such as handouts of each session so you can refer back to them again More handouts of flexibility. More tips and help on how one can include flexibility into our lives, and highlight the benefits of doing so	This category was less presented in the qualitative feedback (two comments) however we included some examples from patients’ feedback: To apply different thinking styles to scenarios during the group and to highlight the consequences of too rigid/flexible thinking More solid ideas about flexibility and ways to be flexible

CRT cognitive remediation therapy

## Discussion

Previous research has found positive feedback and potential benefits of group CRT. Results from this study replicate most of the previous CRT findings within a larger sample. The results indicated that there is a significant improvement on the patients’ self-perceived cognitive flexibility and bigger picture thinking. This is an important finding as patients with AN often have difficulties with their cognitive flexibility and bigger picture thinking [4, 5, 30], which is thought to contribute to the maintenance of the disorder [31].

In addition, consistent with previous literature [24] patients rated their ability to change higher after completing the CRT group, suggesting an increase in the patients’ self-esteem and confidence in the recovery process after taking part in the group. Poor motivation is a common challenge in AN [32], where increased motivation has been shown to predict reduction in eating disorder pathology after treatment [33]. Patients who report high readiness to change are more likely to have a favourable short-term outcome than patients who report low readiness to change [34]. Motivation is recognized as a key factor for translating cognitive changes into actual changes in everyday life [13].

Psychological interventions in a group format have previously presented similar results as individual therapies [35] and can bring unique benefits that are not achievable when working with patients individually. These benefits include sharing experiences and learning from others in a safe and therapeutic environment, as well as being with other people and practicing interpersonal skills. Individuals with AN have difficulties making social contacts [36, 37], as well as reporting high levels of social anhedonia—an absence of pleasure derived from being with people [38, 39]. It has been observed that patients with AN often remain isolated and avoid communicating with other patients in inpatient settings [40]. Thus, CRT in a group format can aid not only cognitive processing, but also social communication.

The positive feedback elicited from patients on the feedback questionnaires highlights the wide acceptability of the treatment. Patients generally found the group positive, and the feedback from the group indicated that the majority of patients found it helpful. In particular, patients liked the fun interactive nature of the group, as well as learning about different thinking styles and how they have an impact on their life. The positive feedback and acceptability of the intervention is promising, as poor treatment engagement is a common problem in existing psychological therapies for AN [41].

This study has some strengths worth mentioning. It is the largest case series reported in CRT group format in AN to date. In addition, the current study for the first time used DFlex as an outcome measure which seems to capture changes in both targeted cognitive areas, for example cognitive flexibility and central coherence (detail vs. bigger picture thinking). Patients’ feedback reported here will inform future studies about patients’ needs and ideas to develop the group further.

This study has a number of limitations worth addressing in future studies. One of the main limitations is the absence of a control condition. It is also important to note that all patients who took part in the CRT groups also received other clinical input during the same time, including other therapeutic groups. It is also worth mentioning that a proportion of patients were weight restored before starting the intervention. As all patients were admitted to a national specialist eating disorder service due to the severity of their disorder, we decided to include participants at all stages of the treatment program. In this study, the relatively late age of onset, comorbid conditions and medication effects were not explored.

In the context of outcome measures, there is always a challenge around which outcomes are most relevant and robust to capture change in the evaluation of psychological work. As CRT predominantly addresses process of thinking, the cognitive domain was identified as the primary outcome in this study. We used DFlex [26] for the first time to evaluate self-perceived cognitive style. Previous literature [22, 24, 42] have used Cognitive Flexibility Scale (CFS) [28] which measures only cognitive flexibility and had a low Cronbach alpha in the current study. Unlike individual CRT work, where CFS has been shown to be sensitive for change [43], this has not been supported in the group evaluation studies, suggesting that CFS may only be effective in assessing change in individual work with longer duration (ten sessions).

Neuropsychological assessments would have been beneficial to evaluate the full effect of group CRT on cognitive variables; this would also aid the comparison between group data and individual format CRT, although it requires resources and time. Patients’ feedback was presented for illustrative purposes in the current study, additional qualitative studies would be beneficial for the further development of the CRT group protocols.

## Conclusion

Group CRT is perceived subjectively effective for patients with AN and results indicate it improves cognitive processes during treatment. The current exploratory case series found improvements in the participants’ self-reported ability to change and provided further ideas for protocol development (e.g. bigger dose, more skills based exercises, homework). Improvement in self-evaluated cognition, confidence and increased motivation are important factors in the recovery journey. Cognitive inefficiencies are one of the maintaining factors of AN, thus patients have appreciated addressing it in parallel with nutritional rehabilitation. Increased confidence and motivation facilitates translation of cognitive changes in everyday life and CRT in group format demonstrates promising findings in this direction.
